# Training multi-layer spiking neural networks with plastic synaptic weights and delays

**DOI:** 10.3389/fnins.2023.1253830

**Published:** 2024-01-24

**Authors:** Jing Wang

**Affiliations:** School of Computer Science and Engineering, University of Electronic Science and Technology of China, Chengdu, China

**Keywords:** spiking neural networks, supervised learning, synaptic weights, synaptic delays, SpikeProp

## Abstract

Spiking neural networks are usually considered as the third generation of neural networks, which hold the potential of ultra-low power consumption on corresponding hardware platforms and are very suitable for temporal information processing. However, how to efficiently train the spiking neural networks remains an open question, and most existing learning methods only consider the plasticity of synaptic weights. In this paper, we proposed a new supervised learning algorithm for multiple-layer spiking neural networks based on the typical SpikeProp method. In the proposed method, both the synaptic weights and delays are considered as adjustable parameters to improve both the biological plausibility and the learning performance. In addition, the proposed method inherits the advantages of SpikeProp, which can make full use of the temporal information of spikes. Various experiments are conducted to verify the performance of the proposed method, and the results demonstrate that the proposed method achieves a competitive learning performance compared with the existing related works. Finally, the differences between the proposed method and the existing mainstream multi-layer training algorithms are discussed.

## 1 Introduction

Deep neural network (DNNs), as a mainstream algorithm of machine learning, has been applied to various fields, such as compute vision (He et al., 2016), speech separation (Subakan et al., 2021), path finding (Arulkumaran et al., 2017), etc. However, the current DNNs suffer from the problem of excessive power consumption, which limit their applications in energy-critical environments (Zhang M. et al., 2021). In contrast, the nervous systems in biological brains require very little energy to handle complex tasks. As a combination of both, the spiking neural networks (SNNs) (Maass, 1997) inherit the existing mature structures and algorithms in the DNNs, and further learn from the way of using spike trains to transmit information between biological neurons. As a result, SNNs have more complex spatial-temporal dynamics and are suitable for ultra-low power devices (Lan et al., 2021; Pan et al., 2021; Zhang et al., 2022). However, at present, there is no training algorithm for SNNs that can give full play to their characteristics, so how to efficiently train SNNs is still an open question.

The current training algorithms for spiking neural networks include training the connection weights between neurons in the network and the delay in the transmission of spike trains between neurons. The first type of training algorithm is consistent with the goal of deep neural networks, which is to enable spiking neural networks to effectively complete tasks by training neural network weights. The training method can be divided into heuristic algorithms, conversion-based algorithms and BP-based algorithms. The representative algorithms of heuristic algorithms are STDP learning algorithms (Caporale and Dan, 2008) and its variants (Yha et al., 2020; Wu et al., 2021c). This class of algorithms is largely based on biological neuroscience findings that when neurons fire together, wire together. The second methods are conversion-based methods (Wu et al., 2021a,b) and their basic idea is to first train a DNN, and then convert the parameters in the trained network to the corresponding SNN through a series of methods. Compared with other state-of-the-art SNN implementations, the inference time and total synaptic operations of the network trained by this method are reduced by at least one order of magnitude. When the length of the simulation time is only eight-time steps, the conversion-based network still achieves good performance in large datasets. The third method is the method based on the surrogate gradient learning (Neftci et al., 2019; Zhu et al., 2021). Although this method can achieve competitive results with DNN in short time steps, this method needs to save the state information of the SNN at each moment. Therefore the computing power and storage requirements are large. The fourth is the event-driven training algorithm (Gütig, 2016; Neftci et al., 2016; Zhang M. et al., 2021; Luo et al., 2022), which only adjusts the network parameters according to the spikes, greatly reducing the training costs.

The above-mentioned learning algorithms for network weights are mostly used for processing static or periodic data and are not effective for fast time-varying signals. The reason is that simply adjusting the synaptic weights cannot effectively extract the rich time-dependent relationship between spike trains in SNNs, while in the biological nervous system, different synapses have various delays in transmitting spike trains (Zhang et al., 2020; Han et al., 2021). In order to further enhence the ability of the SNN model to process fast time-varying data, based on the original synaptic weight training, a training algorithm for the transmission delay between synapses is added.

However, there is biological evidence that the brain's biological synaptic latency is not a constant and there is no uniformity in the rules of latency variation (Sun et al., 2023), so this is also an open area of research. The DL-ReSuMe (Taherkhani et al., 2015a) algorithm is proposed to merge the delay shift approach and ReSuMe-based weight adjustment to enhance the learning performance. After that, Multi-DL-ReSuMe is proposed to train multiple neurons to classify spatiotemporal spiking patterns (Taherkhani et al., 2015b). Shrestha and Orchard (2018) proposes a general backpropagation mechanism for learning synaptic weights and axonal delays which overcomes the problem of non-differentiability of the spike function and uses a temporal credit assignment policy for backpropagating error to preceding layers. Sun et al. (2022) proposes the rectified axonal delay (RAD) as an additional degree of freedom for training that can easily be incorporated into existing SNN frameworks. The new model can perform well on problems where timing matters using very few parameters. DW-ReSuMe (Han et al., 2021) is proposed to achieve a spike train learning task, which is combined with delay learning based on weight training. The RL-Squares-Based Learning Rule (Zhang Y. et al., 2021) is proposed to generate the desired spatiotemporal spike train. The gradient descent-based synaptic delay learning algorithm (Luo et al., 2022) is proposed to improve the sequential learning performance of single-spike neurons.

Although these methods increase the model's ability to process time-series-related data, they need to face the problem of exploding or vanishing gradients in the training process. It is necessary to select the hyper-parameters of the model and algorithm very carefully to make the model converge effectively.

The contribution of this paper includes the following points:

1. This paper introduces synaptic delays between neurons based on the Spike Response Model (SRM) (Gerstner, 1995) model, and combines the SpikeProp (Bohte et al., 2002) algorithm to propose a new learning algorithm for training synaptic delays. This algorithm effectively increases the ability of SNN to deal with fast time-varying tasks.
2. This paper proposes a gradient replacement strategy to effectively reduce the impact of the gradient explosion problem in the training process of the SpikeProp algorithm.
3. In this paper, the proposed training algorithm is applied to several different data sets for testing. The experimental results show that the method presented in this paper effectively increases the ability of SNN to process fast time-varying data.

## 2 Materials and methods

In this section, the basic theoretical knowledge of the neuron model that we used in this paper will be introduced first. Then, we will further introduce the learning algorithm of the SpikeProp. Finally, the proposed learning algorithm is presented and the processing of algorithm derivation is given in detail.

### 2.1 Neuron model

Inspired by the biological brains, spiking neural networks (SNNs) (Maass and Bishop, 2001), which are often referred to as the third generation of artificial neural networks, employ a spike function to replicate the information transfer observed in biological neurons. SNNs possess the unique capacity for biological plasticity, allowing them to encode external inputs into spike trains (Izhikevich, 2003). When these spike trains are processed, they are reduced to two fundamental factors (Pfeiffer and Pfeil, 2018): (1) spike time, which involves the relative timing of pre-synaptic and post-synaptic spikes, and (2) synaptic type, encompassing attributes like excitatory or inhibitory properties and the strength of synaptic connections.

In SNNs, every neuron remains silent until it receives a spike. Once receiving incoming information, each neuron experiences changes in membrane voltage. Output spikes are generated only when the total membrane voltage surpasses the neuron's threshold θ, after which they propagate backward (Ghosh-Dastidar and Adeli, 2009). One widely utilized spiking neuron model is the spike response model [SRM (Gerstner, 1995)]. The membrane voltage in the SRM is calculated as shown in Equation 1:


(1) $$\begin{array}{l} V_{j}^{l+1}\left(t\right)=\underset{i}{\sum }w_{ij}^{l+1}\cdot K\left(t-t_{i}^{l}-d_{i}^{l+1}\right), \end{array}$$

where $V_{j}^{l+1}$ represents the membrane potential of the *j*th neuron in layer *l* + 1, and the $t_{i}^{l}$ is the firing time of the *i*th neuron in layer *l*. $w_{ij}^{l+1}$ and $d_{ij}^{l+1}$ are the synaptic efficacy and delay between these two neurons, respectively. The kernel *K*(·), which determines the shape of postsynaptic potentials (PSPs), is defined as Equation 2:


(2) $$\begin{array}{l} K\left(x\right)=V_{\text{norm}}\left[\exp\left(-\frac{x}{\tau_{m}}\right)-\exp\left(-\frac{x}{\tau_{s}}\right)\right],\;x>0, \end{array}$$

where τ_*m*_ and τ_*s*_ represent the membrane time constant of neurons, respectively. *V*_*norm*_ is the result of PSPs being normalized, which makes the value of PSPs between 0 and 1 and is calculated by Equation 3:


(3) $$\begin{array}{l} V_{\text{norm}}=\frac{\beta^{\beta /\left(\beta -1\right)}}{\beta -1}, \end{array}$$

where β = τ_*m*_/τ_*s*_.

**Figure 1:**
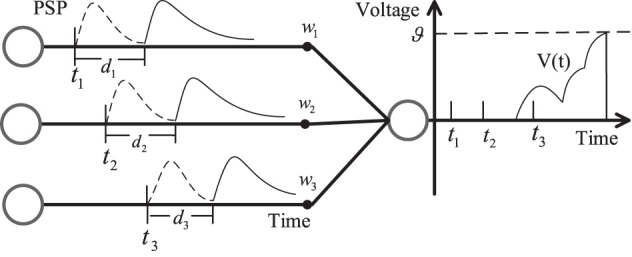
SRM neuron model with synaptic delay.

Figure 1 shows the SRM neuron model with synaptic delay. There are three pre-synaptic neurons with the weight *w*_*i*_ and the delay time *d*_*i*_ (*i* = 1, 2, 3). The delay time ensures the firing time at the synapse is delayed, which makes the membrane potential *V*(*t*) change between *t*_2_ and *t*_3_.

### 2.2 Learning algorithm of the SpikeProp

The SpikeProp algorithm, an improvement upon the traditional backpropagation algorithm for artificial neural networks (ANN), is designed to facilitate the learning process of multi-layer feed-forward spiking neural networks (SNN) (Bohte et al., 2002). Notably, the algorithm imposes a constraint, allowing each neuron to fire at most once within each layer. The SpikeProp algorithm employs the time minimum mean square error function as its error function, which is illustrated in Equation 4:


(4) $$\begin{array}{l} E=\frac{1}{2}\underset{k}{\sum }{\left(t_{k}^{o}-t_{k}^{d}\right)}^{2}, \end{array}$$

where $t_{k}^{o}$ represents the time at which the output neuron *k* emits an actual spike, and $t_{k}^{d}$ denotes its target spike time. SpikeProp utilizes the SRM model, which allows for the derivation of the relationship between firing time and membrane voltage through mathematical analysis. The weight update value is obtained by minimizing the mean square error, as defined in Equation 5:


(5) $$\begin{array}{l} \Delta w_{ij}^{l}=-\eta \frac{\partial E}{\partial w_{ij}^{l}}=-\eta \frac{\partial E}{\partial t_{j}^{l}}\frac{\partial t_{j}^{l}}{\partial V\left(t_{j}^{l}\right)}\frac{\partial V\left(t_{j}^{l}\right)}{\partial w_{ij}^{l}}=-\eta \cdot \delta_{j}^{l}K_{ij}^{l}, \end{array}$$

where $\Delta w_{ij}^{l}$ stands for the gradient of the synaptic weight between *i*th presynaptic neuron in layer *l* − 1 and *j*th postsynaptic neuron in layer *l*. $\delta_{j}^{l}$ is the intermediate quantity for gradient calculation, which can be expressed as $\frac{\partial E}{\partial t_{j}^{l}}\frac{\partial t_{j}^{l}}{\partial V\left(t_{j}^{l}\right)}$, and $K_{ij}^{l}=\frac{\partial V\left(t_{j}^{l}\right)}{\partial w_{ij}^{l}}=K\left(t_{j}^{l}-t_{i}^{l-1}-d_{ij}^{l}\right)$ represents the unweighted postsynaptic potential. η is the learning rate. To simplify notation, we abbreviate $V_{j}^{l}$ as *V*.

However, when the membrane voltage reaches the firing threshold, it promptly resets to the resting potential, resulting in a spike emission at that specific moment, rendering it non-differentiable. Consequently, the direct calculation of $\frac{\partial t_{j}}{\partial V\left(t_{j}^{l}\right)}$ within $\delta_{j}^{l}$ becomes unfeasible. To address this challenge, SpikeProp introduces the concept of a linear hypothesis. This entails assuming that within a sufficiently small neighborhood around $t=t_{j}^{l}$, the membrane potential can be reasonably approximated as a linear function of time, which is defined as:


(6) $$\begin{array}{l} \frac{\partial t_{j}^{l}}{\partial V\left(t_{j}^{l}\right)}=\frac{-1}{\partial V\left(t_{j}^{l}\right)/\partial t}=\frac{-1}{\underset{i}{\sum }w_{ij}^{l}\left(\partial K_{ij}^{l}\left(t_{j}^{l}\right)/\partial t\right)} \end{array}$$

### 2.3 The proposed learning algorithm

SpikeProp primarily focuses on adjusting synaptic weights, which is an essential aspect of biological synaptic plasticity. However, it overlooks another crucial element, synaptic delay plasticity, which imposes limitations on its overall performance and diminishes its biological interpretability. Conversely, in addressing the non-differentiability issue resulting from spike discontinuity, SpikeProp resorts to the approach outlined in Equation 6. Unfortunately, this solution introduces another challenge: the possibility of a gradient explosion due to the rapid membrane voltage change near the firing threshold. In the subsequent sections, we present solutions to these two problems individually.

#### 2.3.1 Learning algorithms with synaptic weights and delay plasticity

To maintain generality, let's assume that the network in question is a multi-layer fully connected network, with the final layer designated as the *o*th layer. Similar to the approach employed in SpikeProp, the network adopts the loss function defined in Equation 4. Subsequently, based on this loss function and employing the error-backpropagation algorithm, the synaptic adjustment rules for layer *l* are formulated as Equation 7:


(7) $$\begin{array}{l} \Delta d_{ij}^{l}\left(w_{ij}^{l}\right)=-\eta_{d\left(w\right)}\frac{\partial E}{\partial d_{ij}^{l}\left(w_{ij}^{l}\right)}, \end{array}$$

where $d_{ij}^{l}\;\left(w_{ij}^{l}\right)$ represents the synaptic delay (weight) of the connection between the *i*th neuron in layer *l* − 1 and the *j*th neuron in layer *l*. η_*d*(*w*)_ represents the learning rate of delays (weights). Since the adjustment of synaptic weights is the same as that of SpikeProp (i.e., Equation 5), next, we only elaborate on the learning of synaptic delay.

- Output layer: For the delay of the output layer $d_{jk}^{o}$, based on the chain rule, we have Equation 8:
(8) $$\begin{array}{l} \frac{\partial E}{\partial d_{jk}^{o}}=\frac{\partial E}{\partial t_{k}^{o}}\frac{\partial t_{k}^{o}}{\partial V\left(t_{k}^{o}\right)}\frac{\partial V\left(t_{k}^{o}\right)}{\partial d_{jk}^{o}}. \end{array}$$
  Similarly, for the convenience of description, we define the intermediate quantity $\delta_{k}^{o}$ as Equation 9:
(9) $$\begin{array}{l} \delta_{k}^{o}=\frac{\partial E}{\partial t_{k}^{o}}\frac{\partial t_{k}^{o}}{\partial V\left(t_{k}^{o}\right)}=\frac{\partial t_{k}^{o}}{\partial V\left(t_{k}^{o}\right)}\cdot \left(t_{k}^{o}-t_{k}^{d}\right), \end{array}$$
  where $\partial t_{k}^{o}/\partial V\left(t_{k}^{o}\right)$ can be solved by Equation 6 like SpikeProp, but due to its drawbacks, we will give an alternative solution later. And the remaining terms in Equation 8 can be computed using Equation 10:
(10) $$\begin{array}{l} \frac{\partial V\left(t_{k}^{o}\right)}{\partial d_{jk}^{o}}=w_{jk}^{o}\cdot \xi_{kj}^{o}, \end{array}$$
  where
(11) $$\begin{array}{l} \begin{array}{ll} \xi_{kj}^{o} & \;=\frac{\partial K\left(t_{k}^{o}-t_{j}^{o-1}-d_{jk}^{o}\right)}{\partial d_{jk}^{o}}\\ =V_{norm}\frac{1}{\tau_{m}}\exp\left(-\frac{t_{k}^{o}-t_{j}^{o-1}-d_{jk}^{o}}{\tau_{m}}\right)\\ -V_{norm}\frac{1}{\tau_{s}}\exp\left(-\frac{t_{k}^{o}-t_{j}^{o-1}-d_{jk}^{o}}{\tau_{s}}\right), \end{array} \end{array}$$
- Hidden layer: For the delay of hidden layer $d_{ij}^{l}$, we have Equation 12:
(12) $$\begin{array}{l} \frac{\partial E}{\partial d_{ij}^{l}}=\frac{\partial E}{\partial t_{j}^{l}}\frac{\partial t_{j}^{l}}{\partial V\left(t_{j}^{l}\right)}\frac{\partial V\left(t_{j}^{l}\right)}{\partial d_{ij}^{l}}=\delta_{j}^{l}\cdot w_{ij}^{l}\cdot \xi_{ij}^{l}, \end{array}$$
  where $t_{j}^{l}$ is the spike time of the *j*th neuron in layer *l*. $\delta_{j}^{l}=\frac{\partial E}{\partial t_{j}^{l}}\frac{\partial t_{j}^{l}}{\partial V\left(t_{j}^{l}\right)}$ with
(13) $$\begin{array}{l} \frac{\partial E}{\partial t_{j}^{l}}=\underset{k}{\sum }\frac{\partial E}{\partial t_{k}^{l+1}}\frac{\partial t_{k}^{l+1}}{\partial V\left(t_{k}^{l+1}\right)}\frac{\partial V\left(t_{k}^{l+1}\right)}{\partial t_{j}^{l}}=\underset{k}{\sum }\delta_{k}^{l+1}\cdot w_{jk}^{l+1}\xi_{kj}^{l+1}. \end{array}$$

To sum up, there are Equation 14:


(14) $$\{\begin{array}{l} \Delta w_{ij}^{l}=-\eta_{w}\cdot \delta_{j}^{l}\cdot K_{ij}^{l},\\ \Delta d_{ij}^{l}=-\eta_{d}\cdot \delta_{j}^{l}\cdot w_{ij}^{l}\xi_{ij}^{l}, \end{array}$$

with


(15) $$\delta_{j}^{l}=\{\begin{array}{ll} \frac{\partial t_{j}^{l}}{\partial V(t_{j}^{l})}\cdot \left(t_{j}^{l}-t_{j}^{d}\right), & l=o\\ \frac{\partial t_{j}^{l}}{\partial V(t_{j}^{l})}\cdot \sum_{k}\delta_{k}^{l+1}w_{jk}^{l+1}\xi_{jk}^{l+1}, & l<o \end{array}$$

While the majority of SNN algorithms traditionally focus solely on updating synaptic weights, we introduce a novel approach by incorporating the adjustment of synaptic delays. This augmentation allows us to achieve joint training of both synaptic weights and delays. This dual-training approach offers two significant advantages: addressing the silent window problem and expanding the parameter space. Silent windows, a common occurrence in spiking neural networks, refer to time periods where no spiking activity takes place, potentially undermining the learning process. Weight updates alone struggle to resolve this issue. However, incorporating delay learning can effectively adjust the distribution of input spikes and mitigate this problem. Moreover, the joint training of both synaptic weights and delays provides a more extensive set of tunable parameters compared to weight-only updates. This expanded parameter space enhances the model's flexibility and can lead to improved overall performance.

#### 2.3.2 Gradient replacement strategy

**Figure 2:**
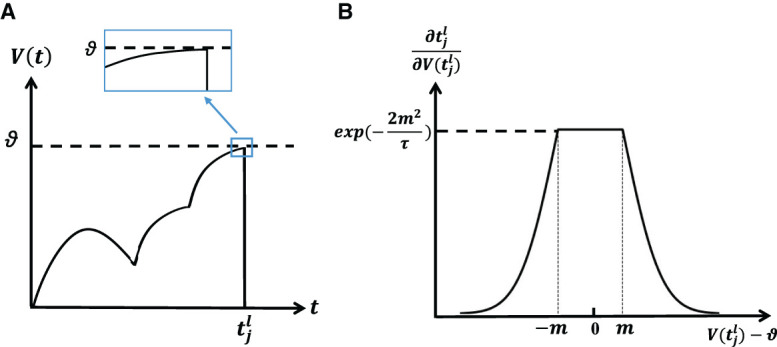
**(A)** Membrane potential reaches the threshold slowly, resulting in the exploding gradient. **(B)** The shape of the surrogate gradient function.

According to the above process, it can be seen that the term $\partial t_{j}^{l}/\partial V\left(t_{j}^{l}\right)$ in Equation 15 is very important for gradient calculation. If its value is calculated according to Equation 6, it means that the derivative of the membrane voltage at the firing time will be critical. More specifically, the analysis of Figure 2A reveals that during a gradual crossing of the membrane voltage threshold, the derivative approaches zero. Consequently, this leads to a significant increase in the magnitude of $\left|\partial t_{j}^{l}/\partial V\left(t_{j}^{l}\right)\right|$, resulting in a phenomenon known as gradient explosion. To address this issue, we draw upon the insights from the rectangle replacement function (Wu et al., 2018), which has demonstrated enhanced convergence in ablation experiments. Building upon this framework, we propose a novel replacement function that mitigates the problem of gradient explosion. As a result, Equation 6 can be replaced by Equation 16, as shown below.


(16) $$\frac{\partial t_{j}^{l}}{\partial V(t_{j}^{l})}=\{\begin{array}{ll} \exp\left(-\frac{2{\left(V(t_{j}^{l})-\vartheta \right)}^{2}}{\tau }\right), & |V(t_{j}^{l})-\vartheta |>m,\\ \exp\left(-\frac{2m^{2}}{\tau }\right), & |V(t_{j}^{l})-\vartheta |\leq m. \end{array}$$

where *m* (0 < *m* < 1) is a constant, and there is currently no accepted theoretical method for finding the optimal *m* on any dataset. But a good way to get an appropriate *m* for a specific task is to use parameter search.

Figure 2B shows the shape of replaced weight. The closer the membrane potential is to the firing time than to the threshold, the larger $\partial t_{j}^{l}/\partial V\left(t_{j}^{l}\right)$, which is consistent with the characteristics of the spike activity. However, it's important to emphasize that this value cannot become infinitely large since it would cause the value of Equation 6 to approach 0, leading the gradient to disappear. In our approach, we impose an upper limit, capping it at exp(−2*m*^2^/τ). This constraint ensures that the influence on $\partial t_{j}^{l}/\partial V\left(t_{j}^{l}\right)$ remains bounded. Actually, the function of the surrogate gradient can be various (Wu et al., 2018).

## 3 Results

In this section, we test the performance of the proposed algorithm on several different datasets, including Iris, Breast Cancer, Liver Disorders, Pima Diabetes, and Ionosphere. By comparison with other algorithms: SpikeProp (Shrestha and Song, 2015), SpikeTemp (Wang et al., 2015), SWAT (Wade et al., 2010), ReSuMe (Ponulak and Kasiński, 2010), SRESN (Dora et al., 2016), and MDL (Taherkhani et al., 2018), the proposed method has a good performance.

**Figure 3:**
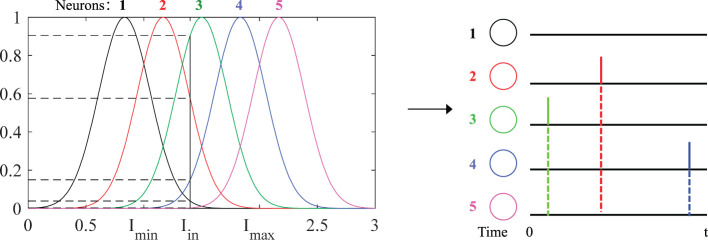
Schematic diagram of population encoding. Each neuron has its own Gaussian receiving function (different colors). *I*_*min*_ and *I*_*max*_ are the minimum and maximum values of the input current. When an input current *I*_*in*_ is fed, the corresponding value on the Gaussian function is its probability value. Finally, these values are encoded into the spiking times distributed in [0, *t*]. The larger probability value corresponds to the earlier spiking time and vice versa, and the spikes will not be emitted if the firing threshold is not reached, such as neurons “1” and “5.”

**Figure 4:**
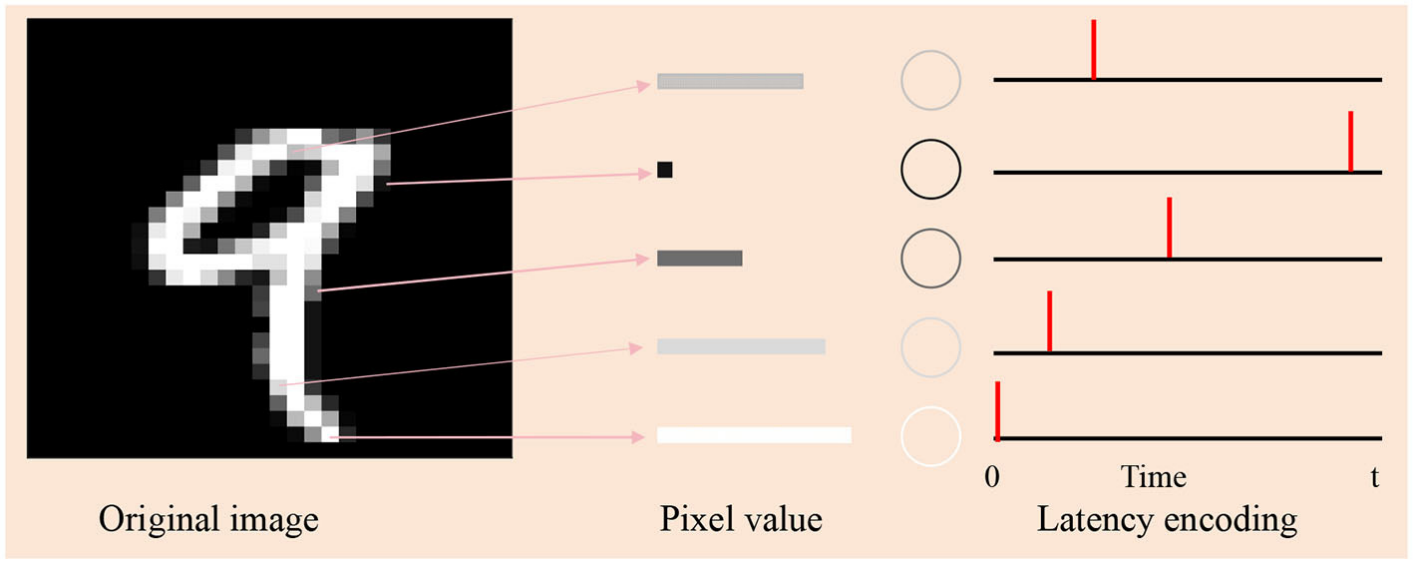
Schematic diagram of latency encoding. The subfigure on the left is an original image of the digit “9” in the MNIST. Its pixel values determine the brightness of each pixel, with higher values corresponding to whiter areas. Depending on the value, we draw five rectangles (in the middle subfigure) of different widths to represent different pixel values, with wider rectangles representing larger values. The right subfigure is the spiking times of the selected five neurons by latency encoding. The larger the pixel value, the earlier the firing time.

The datasets can be divided into two types: real-value datasets and image-related datasets. Samples from these databases cannot be fed directly into the network and need to be encoded into spike sequences. In real-value datasets, the population encoding (Bohte et al., 2002; Shrestha and Song, 2015; Wang et al., 2015; Taherkhani et al., 2018) is used to convert these values to input spike trains, as shown in Figure 3. In image datasets, the latency encoding (Hopfield, 1995; Hu et al., 2013) is used, as shown in Figure 4.

In the output layer each output neuron corresponds to a category, and when a training sample is fed, the corresponding output neuron is trained to fire at desired output spike time *t*_*d*_ generated by the dynamic decoding method (Luo et al., 2019; Zhang Y. et al., 2021), while the other neurons are kept silent.

### 3.1 Classification of Iris dataset

**Figure 5:**
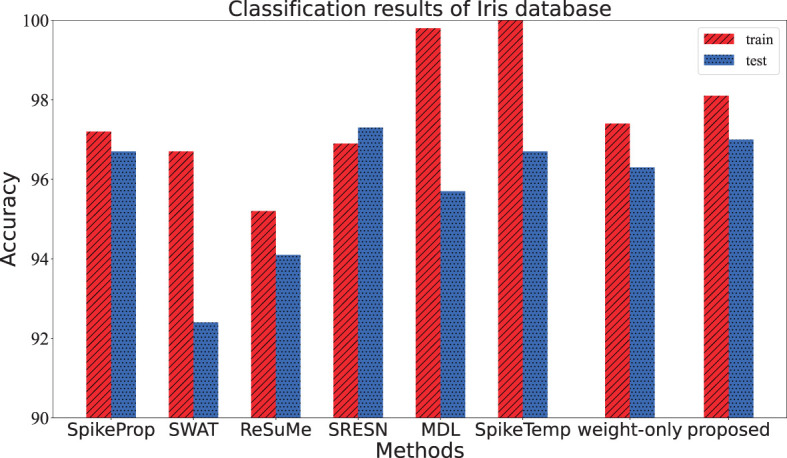
The training accuracy and test accuracy for various methods on Iris database. The best training accuracy of all methods is SpikeTemp, but the best test accuracy is SRESN which is 0.3% higher than the proposed algorithm.

As one of the most well-known pattern recognition databases, it is divided into three categories. Each category has 50 samples with four attributes: sepal length, sepal width, petal length, and petal width. Among them, 25 samples of each category were used as the training set, and the others were used as the test set. The network structure is 25-20-3, and a sample is considered correctly classified if either its target neuron fire the most spikes or the membrane potential of its target neuron is the maximum when none of the output neurons fire. The network architecture, training epochs, train and test accuracy of our works and the contrasting methods are all depicted in Table 1. The contrast of train and test accuracy of all methods are further illustrated in Figure 5. From the results, the proposed method outperforms these methods: SpikeProp, SWAT, MDL, ReSuMe, and SpikeTemp. SRESN achieved the best test accuracy, and the proposed model is only slightly below it.

**Table 1:** Classification results of Iris database.

**Method**	**Architecture**	**Epoch**	**Train (%d)**	**Test (%d)**
SpikeProp	25-10-3	1,000	97.2 (1.9)	96.7 (1.6)
SWAT	24-312-3	500	96.7 (1.4)	92.4 (1.7)
ReSuMe	160-3	200	95.2 (1.4)	94.1 (2.0)
SRESN	24-(6-10)	102	96.9 (1.0)	97.3 (1.3)
MDL	169-360-3	100	99.8 (/)	95.7 (/)
SpikeTemp	120-87	/	100 (/)	96.7 (/)
This work (weight-only)	25-10-3	1,000	97.4 (1.2)	96.3 (1.1)
This work	25-10-3	1,000	98.1 (1.3)	97.0 (1.4)

### 3.2 Classification of Breast Cancer dataset

**Figure 6:**
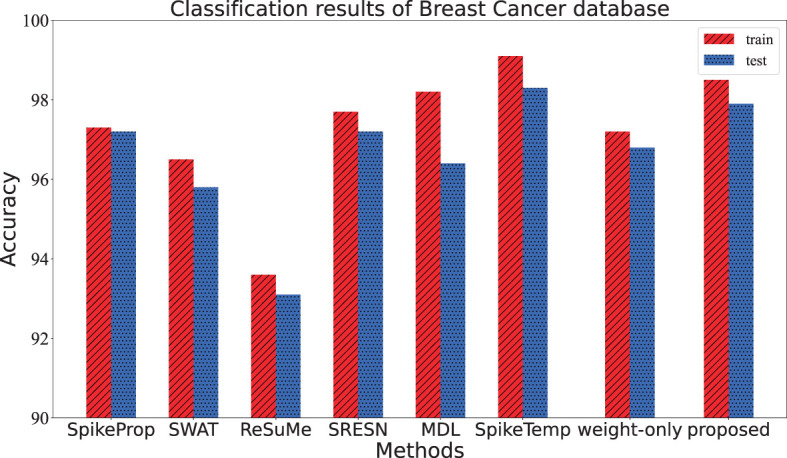
The training accuracy and test accuracy for various methods on Breast Cancer database. The training and test accuracy of the proposed method is the second, slightly lower than SpikeTemp.

The data were collected from clinical studies conducted between January 2014 and December 2014 and were derived from microscopic biopsy images of breast lumps in patients with breast cancer. Each sample has 10 attributes to describe the characteristics of the nucleus of the mass, including radius, texture, perimeter, and so on. It is divided into two types of data: benign and malignant cancers. This dataset has 569 instances, of which 357 are benign and the remaining 212 are malignant. Among them, 179 benign samples and 106 malignant samples make up the training set, and the rest were used as test sets. The network architecture of the proposed method is 55-15-2. Two output neurons correspond to the benign sample and the malignant sample. If the benign output neuron fires the more spikes or has a larger membrane potential than the malignant one, the benign sample was correctly classified; and vice versa. The network architecture, training epochs, train and test accuracy of our works are shown in Table 2, along with those of the other methods. Additionally, the contrast of train and test accuracy of all methods are further illustrated in Figure 6. Experimental results show that this method outperforms most comparison algorithms including SpikeProp, SWAT, ReSuMe, SRESN, and MDL. And our model is only 0.4% lower than SpikeTemp.

**Table 2:** Classification results of Breast Cancer database.

**Method**	**Architecture**	**Epoch**	**Train (%d)**	**Test (%d)**
SpikeProp	55-15-2	1,000	97.3 (0.6)	97.2 (0.6)
SWAT	54-702-2	500	96.5 (0.5)	95.8 (1.0)
ReSuMe	135-2	200	93.6 (0.7)	93.1 (0.8)
SRESN	54-(8-12)	102	97.7 (0.6)	97.2 (0.7)
MDL	/	100	98.2(/)	96.4 (/)
SpikeTemp	135-306	/	99.1 (/)	98.3 (/)
This work (weight-only)	55-15-2	1,000	97.2 (0.9)	96.8 (0.7)
This work	55-15-2	1,000	98.5 (0.8)	97.9 (0.5)

### 3.3 Classification of Liver Disorders dataset

**Figure 7:**
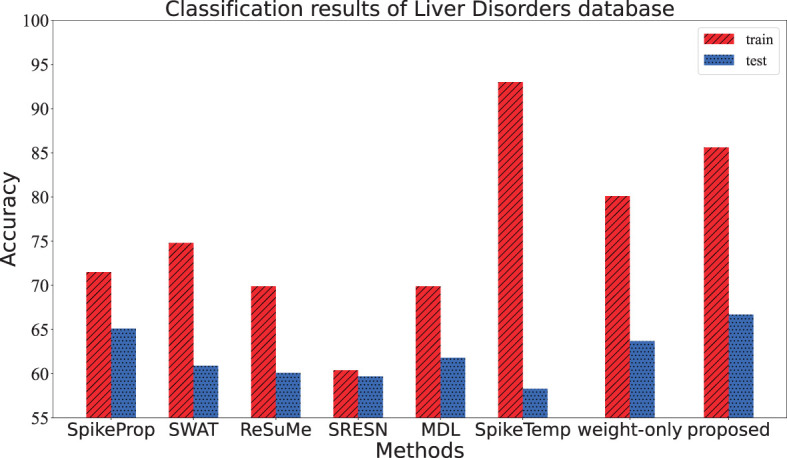
The training accuracy and test accuracy for various methods on Liver Disorders database. The training accuracy of the SpikeTemp is the best, while the test accuracy of the proposed method is the best.

This dataset consisted of 345 samples of seven attributes, amongst which the first five attributes are blood data related to the development of liver disease, and the sixth attribute is the number of alcoholic drinks per day. It is a binary classification, with half of the data in each category comprising the training set and the other half comprising the test set. The input neurons, hidden neurons and output neurons are 37, 15, and 2, respectively. If the target neuron of a sample fires the overwhelming spikes or has the larger membrane potential, this sample is considered to be correctly classified. The performance of the proposed method in Table 3 and Figure 7 comes from the average of 20 trials with 3,000 training epochs, which outperforms other methods in terms of test accuracy.

**Table 3:** Classification results of Liver Disorders database.

**Method**	**Architecture**	**Epoch**	**Train (%d)**	**Test (%d)**
SpikeProp	37-15-2	3,000	71.5 (5.2)	65.1 (4.7)
SWAT	36-468-2	500	74.8 (2.1)	60.9 (3.2)
ReSuMe	150-2	200	69.9 (5.3)	60.1 (3.4)
SRESN	36-(6-9)	715	60.4 (1.7)	59.7 (1.7)
MDL	246-360-2	100	69.9 (/)	61.8 (/)
SpikeTemp	150-226	/	93.0 (/)	58.3 (/)
This work (weight-only)	37-15-2	3,000	80.1 (2.7)	63.7 (2.4)
This work	37-15-2	3,000	85.6 (3.4)	66.7 (3.1)

### 3.4 Classification of Pima Diabetes dataset

**Figure 8:**
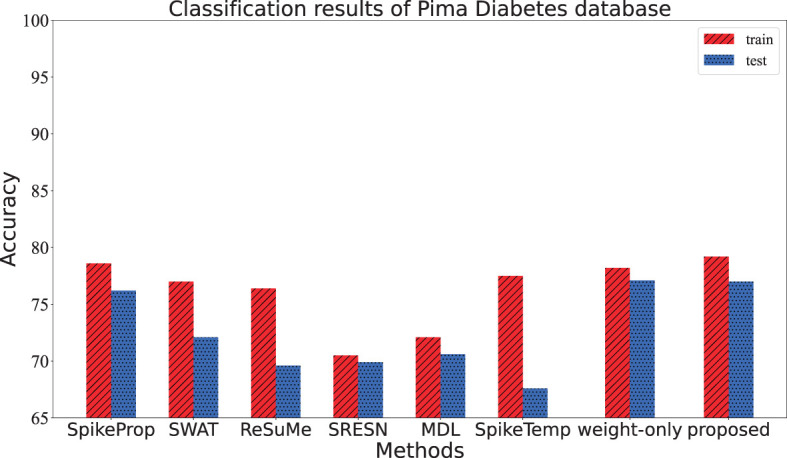
The training accuracy and test accuracy for various methods on Pima Diabetes database. The training accuracy and test accuracy of the proposed method is the best.

This dataset contains women of at least 21 years of Pima Indian ancestry. It is also a binary classification to predict whether a patient has diabetes or not on the basis of eight attributes, including Pregnancy, Glucose, Glucose, etc. The data set includes 768 samples that are divided into training/test sets in a 1:1 ratio. The network structure is 55-20-2, and the conditions for correct classification are as follows: (1) the target output neuron of the input sample fires the most spikes, (2) the membrane potential of the target neuron overwhelms the other one when firing the same spikes. After 20 training trials of 3,000 epochs, the accuracy of our model is shown in Table 4. From Table 4 and Figure 8, the training and test accuracy of the proposed method are much higher than the all the other contrasting methods.

**Table 4:** Classification results of Pima Diabetes database.

**Method**	**Architecture**	**Epoch**	**Train (%d)**	**Test (%d)**
SpikeProp	55-20-2	3,000	78.6 (2.5)	76.2 (1.8)
SWAT	54-702-2	500	77.0 (2.1)	72.1 (1.8)
ReSuMe	80-2	200	76.4 (1.5)	69.6 (2.0)
SRESN	54-(9-14)	254	70.5 (2.4)	69.9 (2.0)
MDL	/	100	72.1 (/)	70.6 (/)
SpikeTemp	80-431	/	77.5 (/)	67.6 (/)
This work (weight-only)	55-20-2	3,000	78.2 (1.7)	77.1 (0.7)
This work	55-20-2	3,000	79.2 (2.0)	77.0 (1.3)

### 3.5 Classification of Ionosphere dataset

**Figure 9:**
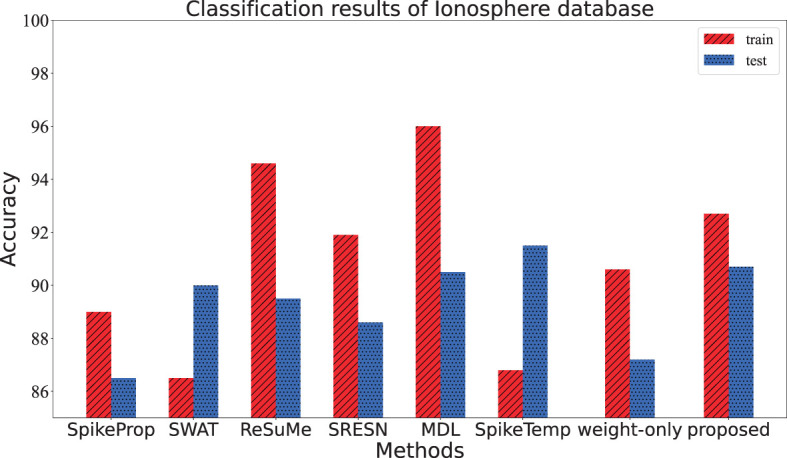
The training accuracy and test accuracy for various methods on Ionosphere database. The proposed method gets the best test accuracy and the smallest gap between training accuracy and test accuracy.

This dataset contains 351 samples of radar data collected by Johns Hopkins University. Each sample consists of 35 attributes, the first 34 attributes are contiguous, and the last attribute is the category label. This data set has two categories, and equal numbers of the samples from each category were added to the training set and test set respectively. The network architecture of this experiment is 205-25-2. When a sample is fed into the network, the category is determined if its target neuron emits the most spikes, or if the target neuron has the maximum membrane potential when emitting the same number of spikes as the other neuron. The network is trained 3,000 epochs in each trial, and the average of 20 trials is shown in Table 5. From Table 5 and Figure 9, the test accuracy of the proposed model ranks only below spikeTemp with a slight gap, but obviously higher than the other five comparison algorithms.

**Table 5:** Classification results of Ionosphere database.

**Method**	**Architecture**	**Epoch**	**Train (%d)**	**Test (%d)**
SpikeProp	205-25-2	3,000	89.0 (7.9)	86.5 (7.2)
SWAT	204-2652-2	500	86.5 (7.2)	90.0 (2.3)
ReSuMe	231-2	200	94.6 (0.6)	89.5 (1.8)
SRESN	204-(16-23)	1,018	91.9 (1.8)	88.6 (1.6)
MDL	/	100	96.0 (/)	90.5 (/)
SpikeTemp	231-223	/	86.8 (/)	91.5 (/)
This work (weight-only)	205-25-2	3,000	90.6 (2.7)	87.2 (1.8)
This work	205-25-2	3,000	92.7 (4.1)	90.7 (2.5)

### 3.6 Classification of MNIST dataset

To exploit and testify the image information learning capacity of the proposed method, we conducted experiments on the widely used MNIST dataset (LeCun et al., 1998), which is a popular choice in deep learning research (Mostafa, 2017; Tavanaei and Maida, 2019; Comsa et al., 2020). This dataset comprises 60,000 training samples and 10,000 testing samples, each of which has a visual scale of 28 × 28 pixels. The pixels are encoded as spike trains through the latency encoding method (Hopfield, 1995) and then fed in the proposed method with the architecture 784-800-10. We compare the experimental results with some effective ANN and SNN works, as detailed in Table 6. The results show that the proposed model has a comparable performance on image data, which outperforms the contrasting SNN models, and only trivially falls behind artificial neural networks in terms of accuracy.

**Table 6:** Comparison with other works on MNIST dataset.

**Method**	**Architecture**	**Learning method**	**Acc (%d)**
Mostafa (2017)	784-800-10	Temporal backpropagation	97.20
Tavanaei and Maida (2019)	784-1000-10	STDP-based backpropagation	96.60
Comsa et al. (2020)	784-340-10	Temporal backpropagation	97.9
ANN (Kheradpisheh and Masquelier, 2020)	784-400-10	Backpropagation with Adam	98.10
S4NN (Kheradpisheh and Masquelier, 2020)	784-400-10	Temporal backpropagation	97.4
This work (weight-only)	784-800-10	Temporal backpropagation	96.7
This work	784-800-10	Temporal backpropagation	97.6

## 4 Discussion

In this paper, we proposed a new supervised learning algorithm for multi-layer spiking neural networks, which considers the plasticity of both synaptic weights and delays. Various experiments are conducted to verify the performance of the proposed learning method, and the experimental results support its superiority. Actually, how to train multilayer spiking neural networks remains an open question. The existing learning rules can be classified as ANN-to-SNN, surrogate gradient method, and spike-driven learning algorithms. In the following, we will compare the proposed method with these methods.

### 4.1 Compared to ANN-to-SNN methods

The ANN-to-SNN methods are proposed to avoid the difficult training of deep spiking neural networks. However, most of the ANN-to-SNN methods are based on the spike rate information, the time information has not been fully leveraged. In addition, the existing ANN-to-SNN conversion methods can only deal with image datasets. Those datasets with rich temporal information like speech and video can not be addressed by these methods. Our algorithm adopts temporal coding to make full use of the time information of spikes and has more potential to process these temporal datasets.

### 4.2 Compared to surrogate gradient methods

Due to the non-differentiable spike function, directly training spiking neural networks is very difficult. To resolve this problem, surrogate gradient-based learning algorithms are proposed. By using a surrogate gradient, these methods do not need to calculate the exact gradients. However, these methods need to do backpropagation at every time step and cost a lot of computing sources. In contrast, our algorithm holds the potential of enabling training and inference in low-power devices.

### 4.3 Compared to spike-driven methods

There are various spike-driven learning methods, such as SpikeProp and STDBP. However, most existing spike-driven learning algorithms only consider the plasticity of synaptic weights and ignore synaptic delay adjustment. In our algorithm, both the synaptic weights and delays are considered adjustable variables to improve both the biological plausibility and the learning performance. Experimental results demonstrate that our algorithm achieves a competitive learning performance compared with the existing related works. In the future, we would like to further extend the application of spike-driven learning algorithms on large-scale datasets and other practical applications (Liu and Li, 2022; Liu et al., 2022a,b).

## Data availability statement

The original contributions presented in the study are included in the article/supplementary material, further inquiries can be directed to the corresponding author.

## Author contributions

JW: Conceptualization, Methodology, Writing – original draft, Writing – review & editing.
